# Experimental Investigation on the Surface Formation Mechanism of NdFeB during Diamond Wire Sawing

**DOI:** 10.3390/ma16041521

**Published:** 2023-02-11

**Authors:** Bin Wu, Zhenyu Zhang, Junyuan Feng, Fanning Meng, Shengzuo Wan, Xuye Zhuang, Li Li, Haoran Liu, Fuxu Zhang

**Affiliations:** 1 Key Laboratory for Precision and Non-Traditional Machining Technology of Ministry of Education, Dalian University of Technology, Dalian 116024, China; 2 School of Mechanical Engineering, Hangzhou Dianzi University, Hangzhou 310018, China; 3 School of Mechanical Engineering, Shandong University of Technology, Zibo 255000, China; 4 Yantai Research Institute and Graduate School of Harbin Engineering University, Yantai 264006, China

**Keywords:** NdFeB, diamond wire sawing, saw marks, surface quality, swing, vibration

## Abstract

Diamond wire sawing is widely used in processing NdFeB rare earth permanent magnets. However, it induces periodic saw marks and fracture chipping pits, which severely affect the flatness and surface quality of the products. In this study, the lateral motion of the diamond wire was monitored to determine the surface formation mechanism. Then, a white light interferometer and an SEM were used to observe the sawed surface profile. Finally, the surface quality was quantitatively studied by identifying the area rate of fracture chipping pits with an image recognition MATLAB script. According to the observation results, the calculation formula of PV which is related to the process parameters was deduced. Additionally, by combining the fracture rate and wire vibration, a novel method was proposed to investigate the optimal process parameters. It can be found that the surface quality sawed at *P* = 0.21 MPa, *v*_f_ = 0.2 mm/min, and *v*_s_ = 1.8 m/s remains better than when sawed at *P* = 0.15 MPa, *v*_f_ = 0.1 mm/min, and *v*_s_ = 1.8 m/s, which means the sawing efficiency can be doubled under such circumstances, i.e., when the surface quality remains the same.

## 1. Introduction

Third generation rare earth magnets NdFeB are extensively used in many fields, such as energy, transportation, mechanical engineering, medicine, and consumer electronics, owing to their superior comprehensive performance [1,2,3,4]. In addition, with the advantages of a higher magnetic energy product, higher coercivity, and better dynamic response characteristics [5,6,7,8], NdFeB magnets can satisfy the current developmental trend of the miniaturization, lightness, and integration of permanent magnet equipment and devices. Consequently, magnets have become an important basic functional material in the industrial development of modern society [9]. Since different product fields have different size requirements for NdFeB magnets, there is a need for sawing sintered NdFeB magnets into different sizes. Traditional NdFeB magnet sawing methods include loose abrasive sawing and diamond disc sawing. Although the efficiency of the disc sawing method is much higher, due to the thicknesses of discs, the material loss during the process is also very high. Moreover, the sawing chips generated during the disc sawing process may create dust pollution, which is a large threat to operator health and the environment. Loose abrasive sawing, though it eliminates the dust pollution problem, is still considered to have a low efficient and high kerf loss. Furthermore, the NdFeB sawing chip, which can be effectively recycled after disc sawing, is mixed with the SiC abrasives during the loose abrasive sawing process. Such a phenomenon makes it difficult to recycle NdFeB chips. When compared to the above methods, DWS is much greener and can greatly reduce kerf loss. As a result, DWS has become the most popular processing method for NdFeB magnets in recent years [10,11,12,13]. However, the periodic saw marks and fracture chipping pits found on the sawed surface have a huge impact on the quality of the products, potentially limiting their application in fields with high precision requirements [14,15,16].

The principle of diamond wire sawing assumes that a high-speed running wire electroplated with diamond abrasive drives into a workpiece, cuts it, and induces material removal. During the process, the trajectory of the diamond wire movement is replicated on the sawed surface as the material is removed. Sawed NdFeB surfaces that are of an insufficient quality need to be ground to meet the requirements of the subsequent electroplating process, which significantly increases the production time and cost. Therefore, in order to achieve improved NdFeB surface quality, it is vital to investigate the surface formation mechanism of the process and analyze the effects of different process parameters on the surface characteristics. 

Much research has been carried out to reveal the complex impact of different process parameters on the diamond wire movement and the quality of the sawed surface. Li et al. [17] studied the relationship between the quality of sawed polycrystalline silicon surface and process parameters, and the results showed that increasing the wire speed or decreasing the feed speed could lead to lower surface roughness. Jia et al. [18] found that the surface roughness tended to decrease and then increase when increasing the feed speed when the wire speed was kept constant. In addition, they also pointed out that there was a unique matching feed speed to attain a lower surface roughness when sawing at different wire speeds. Teomete [19] developed an analytical model for long waviness generation and pointed out that the oblique cutting of the individual grit causes the wire to diverge in the X–Y plane, inducing the long waviness. He also found that surface damage in the case of alumina ceramic was mainly caused by intergranular failure and grain dislodgement [20] and that its depth was independent of tension but increased with an increased feed speed and decreased wire speed [21]. Huang et al. [22,23] discovered that wire vibration is a key factor in terms of the kerf loss. Furthermore, they found more signs of plastic removal on sapphire and SiC wafer when cutting at a higher wire speed. Tang et al. [24] indicated a significant correlation between the wire vibration and the surface roughness of silicon carbide wafer. They determined the mathematical equation for the wire vibration along the feed direction versus the process parameters. The experimental results demonstrated that increasing the feed speed or decreasing the wire speed results in a larger wire vibration amplitude. According to the axially moving string theory proposed by Wickert et al. [25] and Huang et al. [26], Chung et al. [27] resolved the quantitative relationship between the string transverse vibration and the excitation based on Green’s function. They suggested that the amplitude and frequency of the excitation were the critical factors affecting the transverse vibration. Wang et al. [28] provided the equation for the relationship between the vibration of diamond beaded rope in the horizontal direction and the process parameters when sawing granite. They found that the vibration amplitude of the bead rope decreased with the increase in wire speed and tension. Wei et al. [29] modeled the wire longitudinal wire vibration during the free abrasive wire sawing process by considering the position and the amount of excitation applied. It was found that as the amount of excitation increased, the wire vibration amplitude gradually decreased. Liu et al. [14] investigated the relationship between NdFeB surface roughness and feed speed during diamond wire sawing. The results showed that when the feed speed was low, the NdFeB grains were peeled off from the substrate due to the influence of the diamond wire vibration, and more brittle fractures were formed on the surface.

Given the current state of the research, many scholars have investigated the motion of diamond wire under different process parameters. However, there are relatively few studies on the diamond wire sawing of NdFeB magnets, and the interaction mechanism between the wire’s lateral motion and the sawed surface morphology of NdFeB magnets has not been studied in detail, with the resulting formation mechanism of the sawing surface morphology remaining unclear. The effects of process parameters on NdFeB surface morphology are also unknown. Therefore, how to improve the sawing process stability and surface quality in the case of NdFeB magnets is remains elusive. In this study, a series of sawing experiments were conducted with different process parameters, and the lateral motion of the diamond wire was monitored by a laser displacement sensor in both no-load and cutting conditions. The profile characteristics of the NdFeB surface were recorded with a white light interferometer. The surface of the samples was also examined and photographed under a scanning electron microscope (SEM). Subsequently, an image recognition MATLAB script was written to quantitatively analyze the surface fracture rate of the NdFeB samples. Finally, considering the decomposition results in terms of the diamond wire lateral displacement, the formation mechanism of NdFeB surface morphology was analyzed, and the effects of different process parameters on NdFeB surface quality were also summarized.

## 2. Materials and Methods

**Figure 1:**
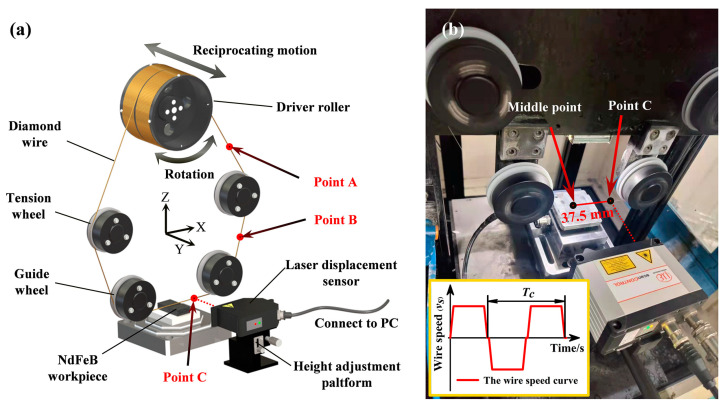
(**a**) Diamond wire sawing experiment setup; (**b**) diamond wire in the sawing area.

The diamond wire cutting machine used in this experiment was produced by Shenyang Kejing Auto-instrument Co., Ltd., Shenyang, China. As shown in Figure 1a, the diamond wire is wound on the driver roller with a certain pitch. The driver roller can rotate and feed along the y-axis to keep the diamond wire in a fixed plane. Figure 1b also presents the motion of the diamond wire. The speed of the driver roller was reduced to 0 rpm during the reversing stopping process, and then the speed of the diamond wire was increased to its maximum in the opposite direction. The forward and reverse motions of the diamond wire constitute a reciprocating cycle with a period of *T*_c_.

During the sawing process, the lateral motion of the diamond wire at points A, B, and C was monitored by an ILD2300-50 laser displacement sensor produced by Micro-Epsilon (Beijing) Measurement Co., Ltd. The laser spot diameter of the ILD2300-50 was 70 μm, which is smaller than the diameter of the diamond wire used in this experiment. The sampling rate of the laser displacement sensor is 10 kHz. By adjusting the height of the micro-positioning platform fixed under the sensor, the laser can be adjusted to the point at the center of the diamond wire. When the energy intensity has only one peak located near the middle position of the map, it is the laser spot is considered to be on the center of the diamond wire. In this experiment, an N35 NdFeB magnet with a size of 25 mm × 10 mm × 50 mm, provided by Advanced Technology & Materials Co., Ltd., Beijing, China, was chosen as a cutting sample. Its composition was provided by the producer, as shown in Table 1. 

The diamond wire involved in sawing, the parameters of which are shown in Table 2, was provided by Guangzhou Shenghai Technology Co., Ltd., Guangzhou, China.

In order to observe the NdFeB micro-structure, a small number of samples were subjected to grinding and polishing before observation. Firstly, the NdFeB magnet was rough ground for 10 min by #600 sandpaper. Secondly, the NdFeB magnet was ground for 15 min with #1200 sandpaper. Then, a polishing slurry with a mass fraction of 5% was prepared using 50 nm SiO_2_ abrasives and distilled water, which was then used in polishing the NdFeB magnet for 30 min. 

To investigate the relationship between the process parameters and the surface morphology of the NdFeB magnet, we selected the process parameters shown in Table 3 for the experiments. According to Liu et al., if the feed speed is too large, the probability of a wire break becomes high. Therefore, in this study, the feed speeds chosen were 0.05 mm/min, 0.1 mm/min, 0.2 mm/min, and 0.3 mm/min [14]. When the driver roller speed was below 200 rpm, the cutting efficiency became too low, resulting in a long sawing time. Therefore, the driver roller speeds selected were 220 rpm, 260 rpm, and 300 rpm, and the corresponding wire speeds were 1.32 m/s, 1.56 m/s, and 1.8 m/s. It was also discovered that the wire undergoes a much higher breaking risk when the tension exceeds 0.23 MPa. According to the parameters in Table 3, the diamond wire sawing no-load experiments and cutting experiments were devised, as shown in Table 4 and Table 5.

After completing the sawing experiments in Table 5, the kerf width was measured under an optical microscope manufactured by Olympus Corporation. According to the method of observing the sample surface adopted by Huang et al. [23], all the samples were observed under a Zygo NewView^TM^ 9000 white light interferometer to obtain the surface profile. 

**Figure 2:**
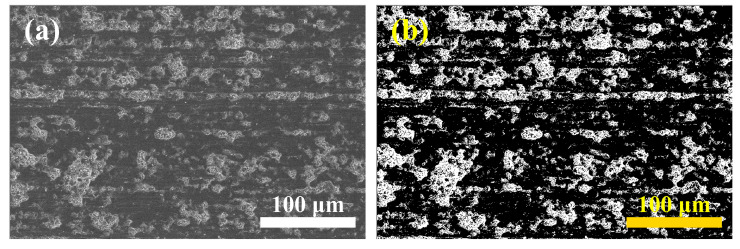
(**a**) The original SEM image of the sawed surface; (**b**) the binarized image after processing by the MATLAB script.

Afterward, an SEM was used to assess the characteristics of the sawed and polished NdFeB surface. Five 380 μm × 330 μm surface regions were randomly chosen and used to calculate the fracture rate, as shown in Figure 2a. The MATLAB script we used to turn the image into a grey image, and then a grey scale threshold of 105 was chosen for the binarization of the image, as shown in Figure 2b. The bright fracture zone pixels were counted and a fracture rate was calculated for each sample. 

## 3. Results and Discussion

### 3.1. Decomposition of the Lateral Motion of the Diamond Wire

**Figure 3:**
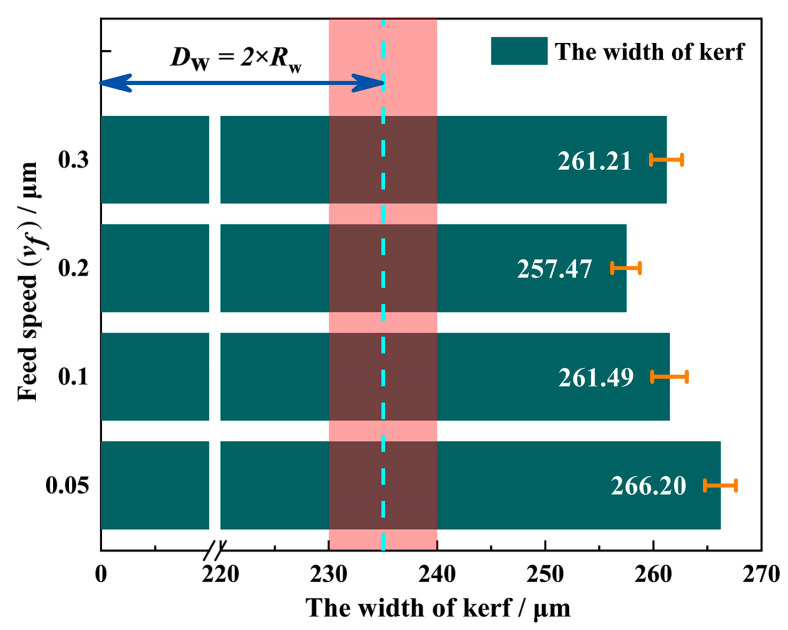
The kerf width sawed at *P* = 0.21 MPa and *v*_s_ = 1.8 m/s.

Figure 3 shows the kerf width at different feed speeds under the process parameters of *v*_s_ = 1.8 m/s and *P* = 0.21 MPa. It can be seen that the kerf width in each group is within the range of 250 to 270 μm.

**Figure 4:**
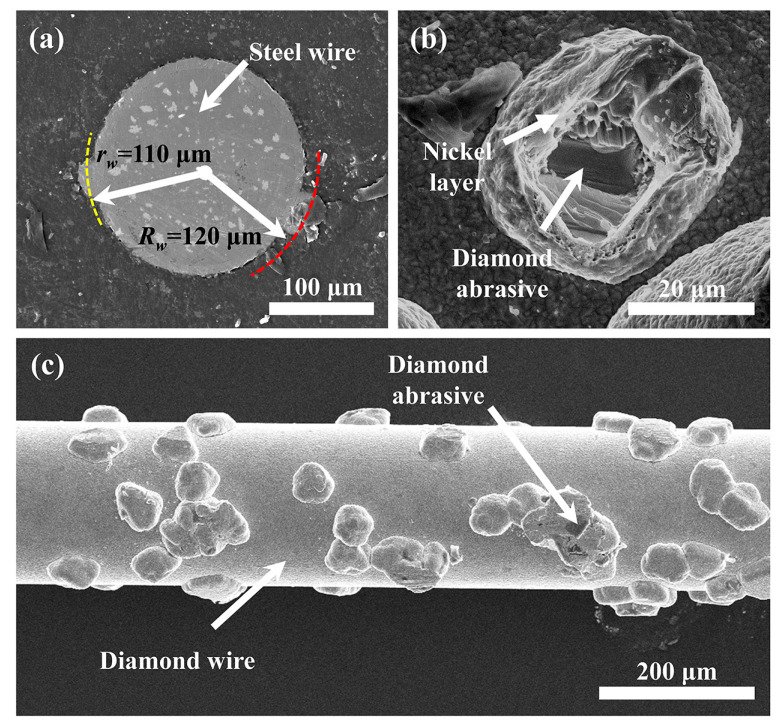
(**a**) Cross-section of the diamond wire; (**b**) diamond abrasive particle; (**c**) a full view of the diamond wire with diamond abrasives.

However, the maximum radius (*R*_w_) of the diamond wire applied in this experiment was around 120 μm, and the effective cutting radius (*r*_w_) was approximately 110 μm, as shown in Figure 4. Therefore, the actual kerf width is at least 20 μm larger than the maximum outer diameter of the diamond wire, which indicates that the diamond wire not only moved along the cutting direction but also along the lateral direction during the sawing process.

**Figure 5:**
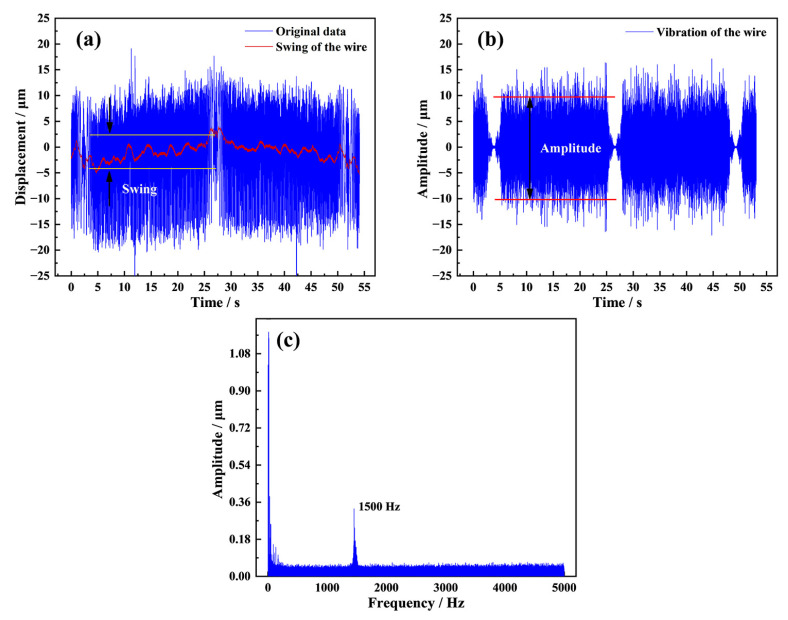
(**a**) Lateral displacement of the diamond wire in one reciprocating cycle; (**b**) the vibration of the diamond wire in one reciprocating cycle; (**c**) the frequency–amplitude characteristics of the wire vibration.

The original motion data of the diamond wire at point C recorded by the laser displacement sensor in the sawing process is shown in Figure 5a. It can be seen that the diamond wire displays a swing motion feature for a period of 48 s during one reciprocating cycle. The frequency–amplitude characteristics of the wire vibration are shown in Figure 5c, and it can also be noticed that the diamond wire vibrates in a lateral direction with a relatively high frequency of 1.5 kHz. Liu et al., in their study [14], obtained the vibration component by subtracting the swing data from the original data, and they also pointed out that wire vibrations have a much higher vibration frequency according to frequency domain analysis. However, in this work, we used a different method to separate two vibration components. Firstly, the original data was fitted by second-order polynomial using a Savitzky–Golay filter to obtain the swing of the diamond wire in one reciprocating cycle, as shown in Figure 5a. Then, a high pass filter with a frequency threshold of 1500 Hz was applied to the original data to obtain the vibration shown in Figure 5b. Finally, the motion of the diamond wire was decomposed into a low-frequency swing and a high-frequency vibration. As seen in Figure 5a, the swing period is consistent with the reciprocating cycle, and the vibration correlates with the speed of the diamond wire. When the diamond wire moves along the axial direction at a uniform speed, the vibration amplitude remains consistent at around 20.5 μm. 

**Figure 6:**
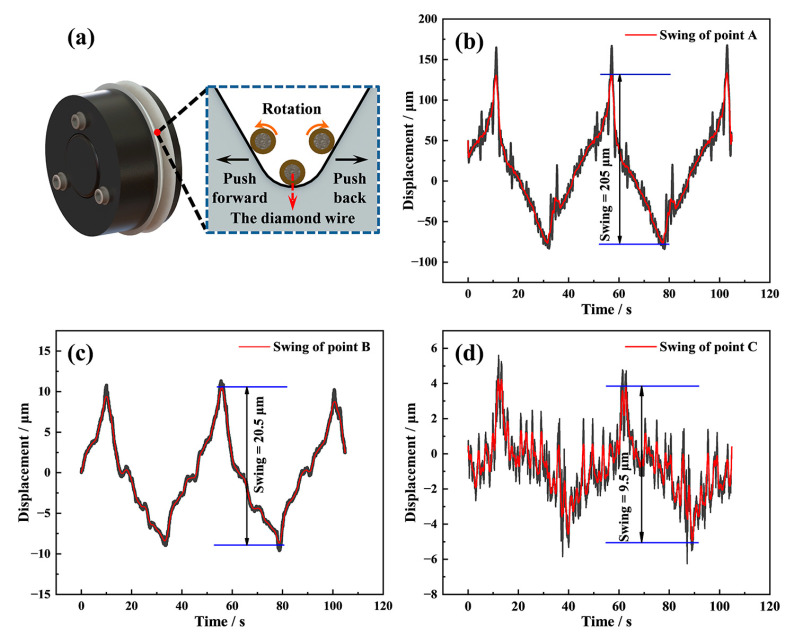
(**a**) Rotation of the diamond wire in the groove bottom; (**b**) the wire swing of point A; (**c**) the wire swing of point B; (**d**) the wire swing of point C.

To explore the cause of the swing, the diamond wire motion was studied under no-load conditions. Firstly, we utilized the laser displacement sensor to monitor the motion of the diamond wire at point A, and Figure 6b shows its motion at this position. As the driver roller moves to the front limit position, the diamond wire reaches the maximum positive offset position. When the driver roller rotates counterclockwise and rewinds to the back limit position, the diamond wire also reaches the maximum negative offset position. The movement of the driver roller caused the swing of the diamond wire to have a larger amplitude of 205 μm at point A.

The lateral displacements of the diamond wire at points B and point C were also monitored to study the transfer of the swing motion caused by the driver roller. In Figure 6c, the displacement of the diamond wire at point B shows the same movement trend and period as point A, but the swing amplitude decreases from 205 to approximately 20.5 μm after running through the tension wheel. In Figure 6d, the diamond wire at point C also exhibits the same periodic swing characteristics as points A and B, but the amplitude further decreases from 20.5 μm at point B to approximately 9.5 μm after going through the guide wheel.

Such a rapid decrease in the lateral swing amplitude is only possible thanks to the restrain from the sidewalls of the V-shaped groove, the structure of which is shown in Figure 6a. The angle of the V-shaped groove is 60°, the radius of the bottom arc is approximately 0.4 mm, and the width of the arc is 6 mm. When the diamond wire passes through the tension wheel groove, the lateral swing of the diamond wire is obstructed, resulting in a significant reduction in the swing amplitude. When the diamond wire goes through the guide wheel groove, the swing of the diamond wire is also restrained, which causes the swing amplitude to reduce again. However, the reduction in this case was significantly lower than the decrease in the swing amplitude from point A to point B. As shown in Figure 6a, the radius of the arc at the bottom of the V-shaped groove is slightly larger than the outer diameter of the diamond wire. Qiu et al. [30] found that the non-co-planar phenomena of the driver roller and wheels caused a certain torsion in the diamond wire transmission process. This promotes the diamond wire rolling along the bottom of the V-shaped groove to have a period equal to that of the reciprocating motion. Therefore, even if the guide wheel grooves restrain the diamond wire swing at point C, it persists regardless.

**Figure 7:**
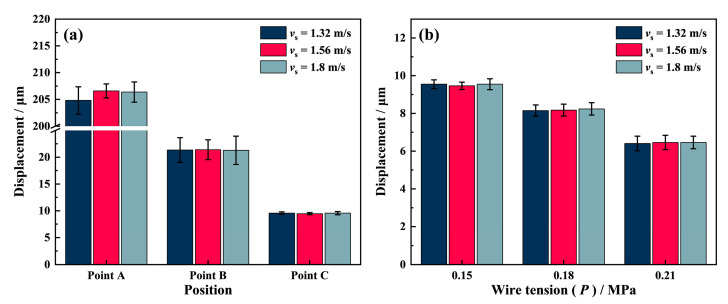
(**a**) The lateral displacement of the diamond wire at different points; (**b**) the lateral displacement of the diamond wire at point C under different wire tension conditions.

To investigate the effects of different process parameters on the swing amplitude, we monitored the diamond wire motion using the laser displacement sensor during no-load conditions and cutting conditions. In Figure 7a, the swing amplitude of point A remains near 205 μm, and those of point B and point C remain near 20.5 μm and 9.5 μm for three different wire speed conditions, respectively. The swing amplitude of the diamond wire at different positions does not vary significantly with the wire speed, indicating that the wire speed has little effect on the swing amplitude under no-load conditions.

The impact of wire tension on the diamond wire swing at point C is particularly significant, as shown in Figure 7b. With the increase in wire tension, the swing amplitude of the diamond wire decreases from 9.5 μm to 6.3 μm. The higher wire tension can cause the diamond wire to be tightly pressed at the bottom of the V-shaped groove, as shown in Figure 6a, so that it is more difficult for it to roll along the bottom arc of the groove under no-load conditions, with this result being consistent with the work of Qiu et al. [30]. Eventually, this leads to a reduction in the diamond wire swing amplitude. 

The kerf loss widths of the samples are approximately 20 to 40 μm, which is much larger than the swing amplitude of the diamond wire. According to the motion decomposition shown in Figure 5, the amplitude of the wire vibration can reach 20.5 μm and occupies the dominant portion in terms of lateral displacement. This therefore indicates that the diamond wire vibration is also an important cause of kerf loss.

In previous studies, the diamond wire was considered as a free string that was fixed at the two guided wheels. During the sawing process, the normal force generated by the cutting action on each abrasive served as the excitation on the string and caused the wire vibration. Therefore, the wire tension is regarded as an important parameter that affects the wire vibration. Wickert et al. [25] and Huang et al. [26] proposed the classical vibration theory that reveals that vibration causes of axially moving continua. It can be expressed as Equation (1):

(1) $$u_{\mathrm{tt}}+2vu_{\mathrm{xt}}-\left(1-v^{2}\right)u_{\mathrm{xx}}=f$$

where *u* is the dimensionless transverse displacement of the wire, *v* is the axial wire speed, *f* is the dimensionless external excitation force, *x* is the spatial coordinate, and *t* is the time coordinate. Chung [27] presented the solution of the wave equation for transversal wire vibration based on Green’s function as Equation (2):

(2) $$\begin{array}{l} u=f_{0}\sum_{n=1}^{\infty }\frac{1}{n\pi }\frac{1}{\omega_{n}^{2}-\omega^{2}}\sin\left(n\pi x_{0}\right)\sin\left(n\pi x\right)[\left(\omega_{n}+\omega \right)\sin\left(\omega t+n\pi v\left(x-x_{0}\right)\right)\\ +\left(\omega_{n}-\omega \right)\sin\left(\omega t-n\pi v\left(x-x_{0}\right)\right)-2\omega \sin\left(\omega_{n}t+n\pi v\left(x-x_{0}\right)\right)] \end{array}$$

where *ω*_n_ = *nπ*(1 − *v*^2^), *ω*_n_ is the dimensionless natural frequencies of the wire, *ω* is the dimensionless frequency of the external excitation, and *f*_0_ is the amplitude of the dimensionless excitation force. Based on this theory, Wang et al. [28] investigated the vibration of a diamond beaded rope during the sawing of granite. The study showed that the transverse displacement decreases with the diamond bead rope tension under the same excitation amplitude and frequency. It can also be seen in Figure 5b that the vibration is directly related to the wire speed. As the wire speed increase, the number of diamond abrasives involved in cutting per unit of time also increases, resulting in a higher excitation frequency. When the feed speed remains constant, the cutting depth of the diamond abrasive decreases with the wire speed, and, as a result, the excitation amplitude also decreases. Since the wire speed is directly related to the excitation amplitude and frequency, its influence on the diamond wire vibration is relatively complicated.

### 3.2. Wire Swing and Surface Profile

**Figure 8:**
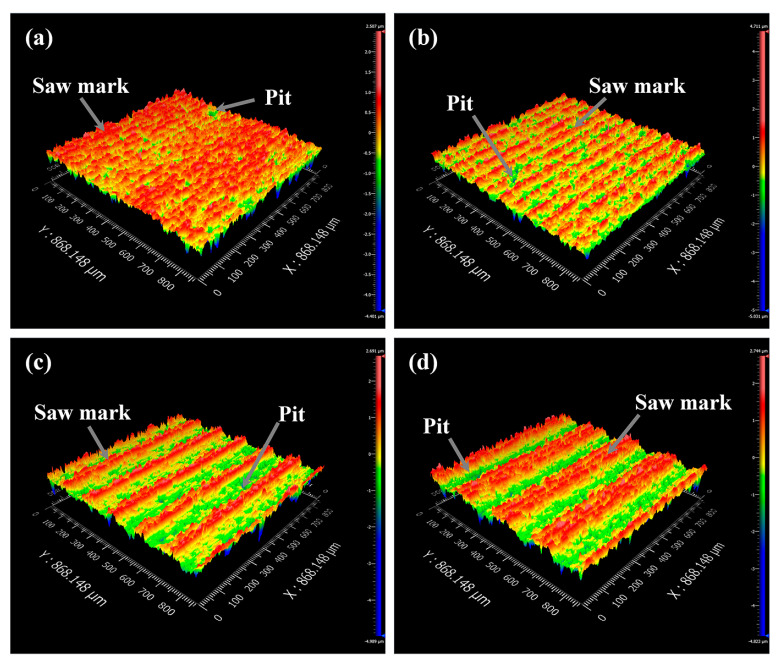
Surface morphology of the sawed surface at *P* = 0.18 MPa, *v*_s_ = 1.8 m/s with a feed speed of: (**a**) *v*_f_ = 0.05 mm/min; (**b**) *v*_f_ = 0.1 mm/min; (**c**) *v*_f_ = 0.2 mm/min; and (**d**) *v*_f_ = 0.3 mm/min.

Figure 8a–d exhibit the surface profile of NdFeB samples sawed at different feed speeds. Saw marks appear on all samples, but the number of saw marks on the surfaces of samples decreases with the feed speed.

The diamond wire sawing platform in this work uses a diamond wire of a certain length, *L*, and saws the workpiece by reciprocating movement. At a given wire speed, *v*_s_, the time, *T*_c_, required for one reciprocating cycle is given by Equation (3) [23]:

(3) $$T_{c}=\left(\frac{2\cdot L}{v_{s}}\right)$$

The theoretical cutting depth, *T*_t_, in one cycle can be expressed as Equation (4):

(4) $$T_{t}=T_{c}\cdot v_{f}$$

According to the theoretical and measured values in Table 6, it can be seen that the period of saw marks is almost the same as the theoretical cutting depth within a reciprocating cycle, with the relative error between them being less than 1.63%. This indicates that the saw marks on the NdFeB surface are related to the reciprocating cycle of the diamond wire, which is consistent with the results of the work of Teomete et al. [19]. Their study was an important basis for studying the influence of process parameters on the mechanism of saw marks on sawed NdFeB surfaces in this work.

**Figure 9:**
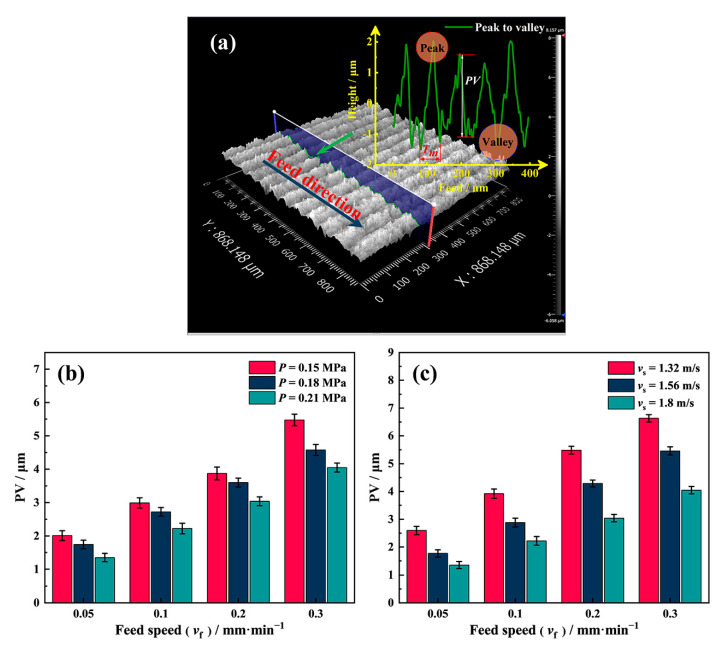
(**a**) Schematic illustration of the PV measurement; (**b**) the effect of the wire tension on the PV value, and (**c**) the effect of the wire speed on the PV value.

Figure 9 shows the effect of different process parameters on the peak-to-valley (PV) values of the saw marks.

In Figure 9b, the PV value of the saw marks reaches the minimum at *v*_f_ = 0.05 mm/min and increases along with the feed speed. The influence of the wire tension on the PV value in Figure 9b shows that the PV value decreases with the tension, following the same trend as the impact of the tension on the diamond wire swing under no-load conditions. The PV value is the lowest when *P* = 0.21 MPa for the same feed speed condition. Keeping the tension constant at 0.21 MPa, the relationship between different wire speeds and PV values is given in Figure 9c. For the surface of the NdFeB samples sawed by four groups of different feed speeds, the PV values are the smallest when *v*_f_ = 1.8 m/s, and the PV values of each group universally show an increasing trend as the wire speed decreases.

**Figure 10:**
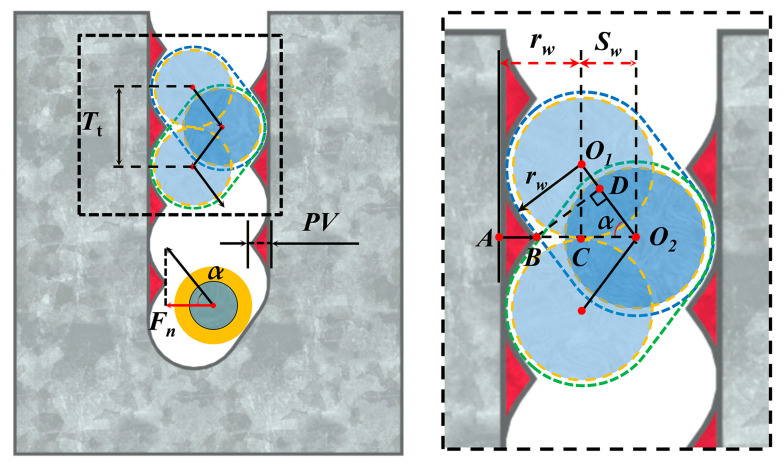
The formation process of periodic saw marks.

Figure 10 reveals the formation mechanism of the wavy saw marks on the sample surface. As the driver roller rotates clockwise and moves forward, the diamond wire is pushed away from the median surface formed by the guide wheel grooves in the positive direction of the y-axis. This movement of the diamond wire is replicated on the NdFeB sample during the sawing process and induces material removal in the area marked in blue, as shown in Figure 10. While the driver roller rotates counterclockwise, the diamond wire is pulled back to its original position, resulting in the material removal process marked in green. The red area in Figure 10 is the residual area after such a reciprocating swing, becoming the saw marks we observed. As the NdFeB magnet continuously feeds, the periodic swing of the diamond wire is continuously reflected on both sides of the kerf. Eventually, a wavy feature with interlocking peaks and valleys is formed on the sample surface. The theoretical formula of the PV value established according to Figure 10 is shown in Equation (5).

(5) $$PV=L_{\mathrm{AB}}=r_{w}+S_{w}-L_{{\mathrm{BO}}_{2}}$$

The *S*_w_ in Equation (5) represents the swing amplitude of the diamond wire, while the length of BO_2_ can be expressed as:

(6) $$L_{{\mathrm{BO}}_{2}}=\frac{L_{\mathrm{BD}}}{\sin\alpha }$$

According to the trajectory of the diamond wire, the length of BD can be expressed as:

(7) $$L_{\mathrm{BD}}=r_{w}$$

Based on the geometric relationship, the swing angle of the diamond wire during the cutting process can be expressed by Equation (8):

(8) $$\alpha =\arctan\frac{L_{O_{1}C}}{L_{O_{2}C}}=\arctan\frac{L_{O_{1}C}}{S_{w}}$$

O_1_C in Figure 9 represents the cutting depth of the diamond wire during half a reciprocating cycle, so the length of O_1_C can be expressed by Equation (9):

(9) $$L_{O_{1}C}=\frac{T_{t}}{2}$$

Following Equation (3) through to Equation (9), the PV value of the saw marks on the NdFeB sawed surface can be determined as Equation (10):

(10) $$PV=S_{w}+r_{w}\cdot \left(1-\frac{1}{\sin\left(\arctan\left(T_{t}/2S_{w}\right)\right)}\right)$$

According to Equation (10), the PV value of the sample surface decreases with the diamond wire speed and increases with the feed speed. This explains the variation in the PV values with the wire speed and feed speed in Figure 9b,c.

**Figure 11:**
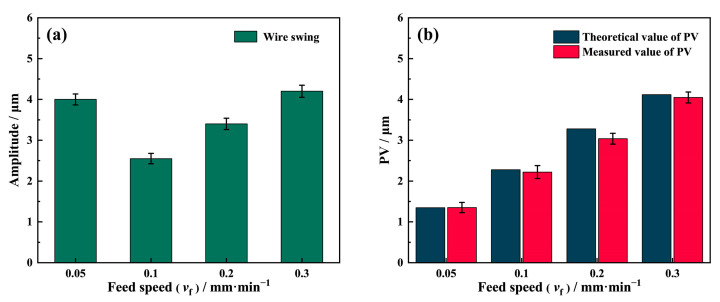
(**a**) Wire swing and (**b**) the comparison of theoretical and measured PV values.

As a main cause of the surface saw mark, the swing amplitude actively affects the PV value of the sawed surface. It is worth noting that the actual swing amplitude of the diamond wire is not consistent with the one under no-load conditions, which is due to the obstruction of the NdFeB magnet during the sawing process. Therefore, the swing amplitude during sawing was recorded by the laser displacement sensor, and the theoretical PV value of the saw marks was calculated using Equation (10). Figure 11b shows that the theoretical PV values are similar to the actual measured values, confirming the theory of the saw marks formation mechanism.

As shown in Figure 11a, the diamond wire swing amplitude during different feed speed sawing is lower than the swing amplitude under the no-load condition. Remarkably, the swing amplitude is at its lowest when the feed speed is 0.1 mm/min. The swing amplitude at *v*_f_ = 0.05 mm/min is much larger than *v*_f_ = 0.1 mm/min, with this being almost the same as at *v*_f_ = 0.3 mm/min. As the feed speed exceeds 0.1 mm/min, the swing amplitude increases with the feed speed. According to Liu et al. [14] and Jia et al. [18], there is an optimal matching relationship between the wire sawing efficiency along the feed direction and the feed speed. When the feed speed is 0.05 mm/min, the wire sawing efficiency is close to the feed speed; hence, the obstruction of the NdFeB sample with regard to the wire swing is weak. It can also be seen from the previous section that the kerf width of the 0.05 mm/min group is much larger than that of other groups, showing that the wire can move freely inside the wide kerf, resulting in a much larger wire swing amplitude. When the feed speed exceeds 0.1 mm/min, the wire sawing efficiency in the vertical direction is much lower than the feed speed. As the feed speed rises, the force, *F_n_*, exerted on the wire by the slope at the kerf bottom in Figure 10 also increases, raising the wire swing amplitude in the horizontal direction. As a result, the swing decreases and then increases with the feed speed.

### 3.3. Wire Vibration and Surface Quality

**Figure 12:**
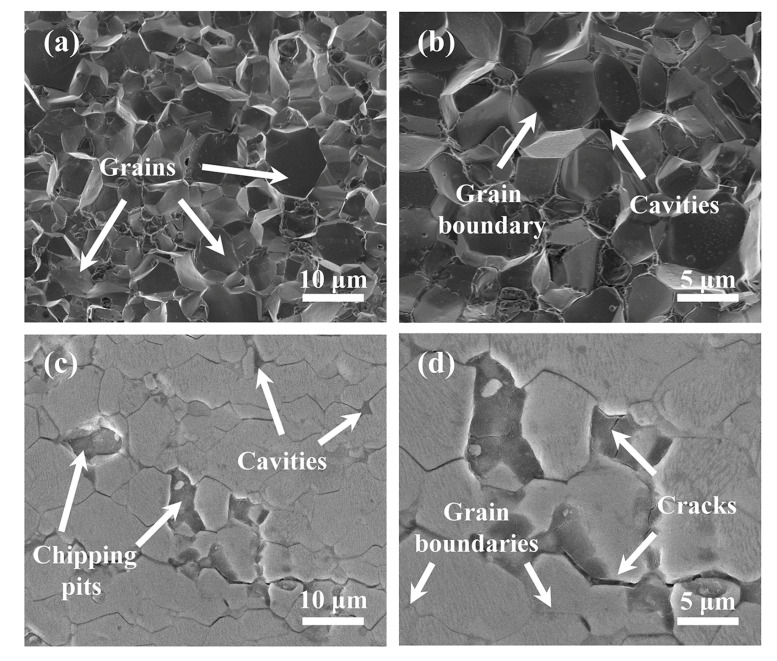
(**a**,**b**) SEM images of the internal organization of NdFeB; (**c**,**d**) SEM images of the polished NdFeB surface.

The classical vibration theory proposed by Wickert et al. [25] points out that excitation is an important factor affecting the transverse displacement of axially moving strings. In the wire saw process, the excitation mainly originates from the random contact between the diamond abrasives and the workpiece. Therefore, not only can the process parameters directly related to the excitation influence the excitation but so can the material’s properties. NdFeB is a soft and brittle material [31] that is mainly manufactured by powder sintering, but it can also be produced by sintering, polymer bonding, and hot deformation [32]. Its hardness is lower than most hard and brittle crystalline materials such as monocrystalline silicon and SiC. As shown in Figure 12a,b, NdFeB magnets are composed of thousands of micrometric-sized grains with a pomegranate-like microstructure, including the NdFeB phase, the B-rich phase, and the Nd-rich phase, which acts as a binding phase [33,34,35]. Due to the very soft and chemically active nature of the Nd-rich phase, the intergranular toughness is very weak. As a result, fractures always appear at the grain boundaries during processing, which will lead to severe damage on the surface of the NdFeB [14].

The surface of the polished NdFeB magnet is shown in Figure 12c,d, where many small cavities and cracks can be seen. The grains can be easily peeled off from their position, forming large fracture chipping pits due to the low intergranular fracture toughness. Such a brittle fracture behavior is further promoted by existing cracks and small cavities [14,36,37]. During the sawing process, diamond abrasives electroplated on the wire randomly strike and scratch the NdFeB to remove the material from the contact area. When the diamond abrasive passes through the cavities, the larger impact will lead to the removal of adjacent grains in a brittle chipping mode. As a result, the process of removing NdFeB material can be regarded as an interrupted process. Afterwards, a sudden change in the cutting force during the fracture process leads to the excitation of the wire vibration.

**Figure 13:**
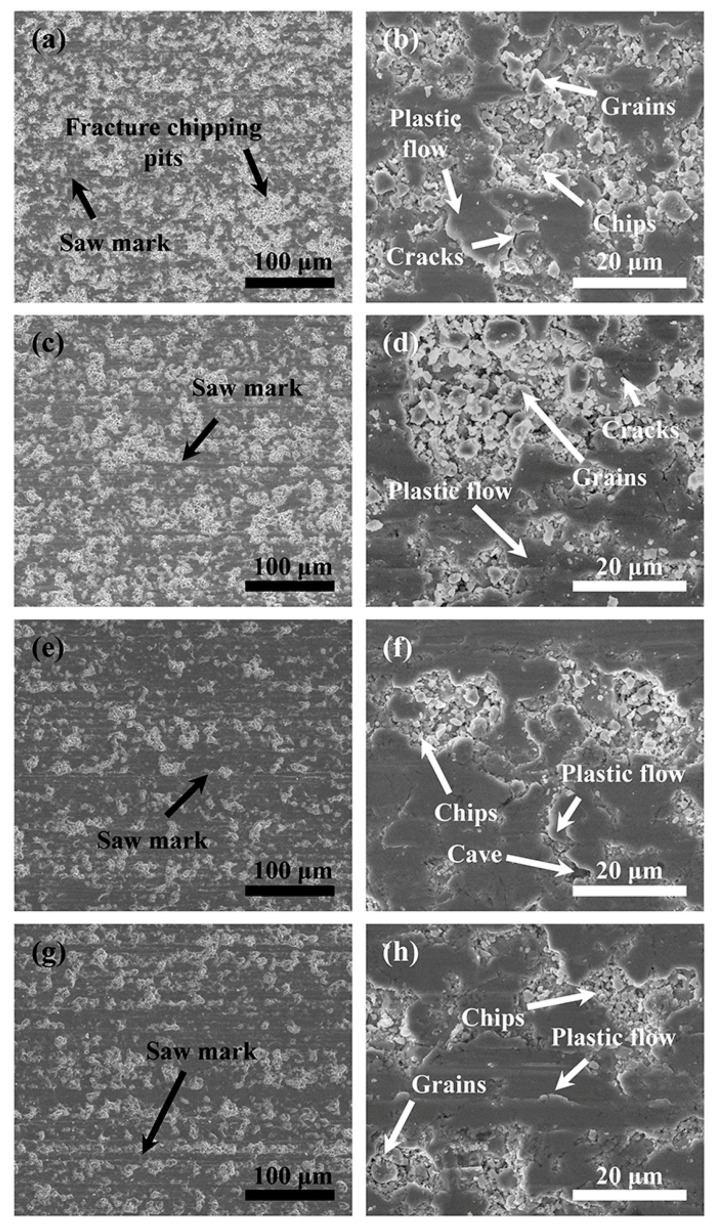
SEM images of the surface of samples sawed at different feed speeds at *P* = 0.21 MPa, *v*_s_ = 1.8 m/s: (**a**,**b**) *v*_f_ = 0.05 mm/min; (**c**,**d**) *v*_f_ = 0.1 mm/min; (**e**,**f**) *v*_f_ = 0.2 mm/min; and (**g**,**h**) *v*_f_ = 0.3 mm/min.

Figure 13 shows an NdFeB surface sawed at different speeds when *P* = 0.21 MPa and *v*_s_ = 1.8 m/s. There are flat areas formed by the plastic cutting mode on all four groups of sawed surfaces. Many fracture chipping pits of different sizes can be found around them. There are also a number of NdFeB chips distributed inside the fracture chipping pits. When *v*_f_ = 0.05 mm/min and *v*_f_ = 0.1 mm/min, the area of fracture chipping pits on the sawed surface is significantly larger than when *v*_f_ = 0.2 mm/min and *v*_f_ = 0.3 mm/min. As can be seen from Figure 13b,d,f,h, more cracks and fracture pits can be observed scattered on the sawed surface.

**Figure 14:**
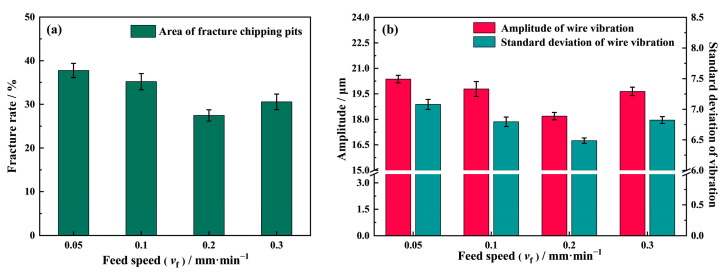
(**a**) Effect of the feed speed on the number of fracture chipping pits; (**b**) the amplitude of the wire vibration and the standard deviation (STD) of the wire vibration at *P* = 0.21 MPa, *v*_s_ = 1.8 m/s.

To quantitatively study the relationship between the different process parameters and the sawed surface quality, a MATLAB image recognition script was written and used to identify and calculate the area percentages of the fracture zones. Figure 14a presents the results under different feed speeds. It can be seen that the fracture rate is its largest at *v*_f_ = 0.05 mm/min, reaching 37.77%. As the feed speed increases to 0.2 mm/min, the fracture rate decreases to 27.45%. However, when the feed speed reaches 0.3 mm/min, the fracture rate increases again to 30.57%. 

In Figure 14b, it can be seen that the wire vibration amplitude trend is consistent with the fracture rate variation in Figure 14a. The vibration amplitude turns at *v*_f_ = 0.2 mm/min when the amplitude is the smallest. The standard deviation (STD) of the diamond wire vibration was used to indicate the homogeneity of the amplitude, as shown in Figure 14b. It can be seen that the wire vibration is relatively more random at *v*_f_ = 0.05 mm/min, and the vibration amplitude is also at its largest. The large vibration amplitude and the high vibration STD show that the interaction between the abrasives and the surface is at its most severe.

**Figure 15:**
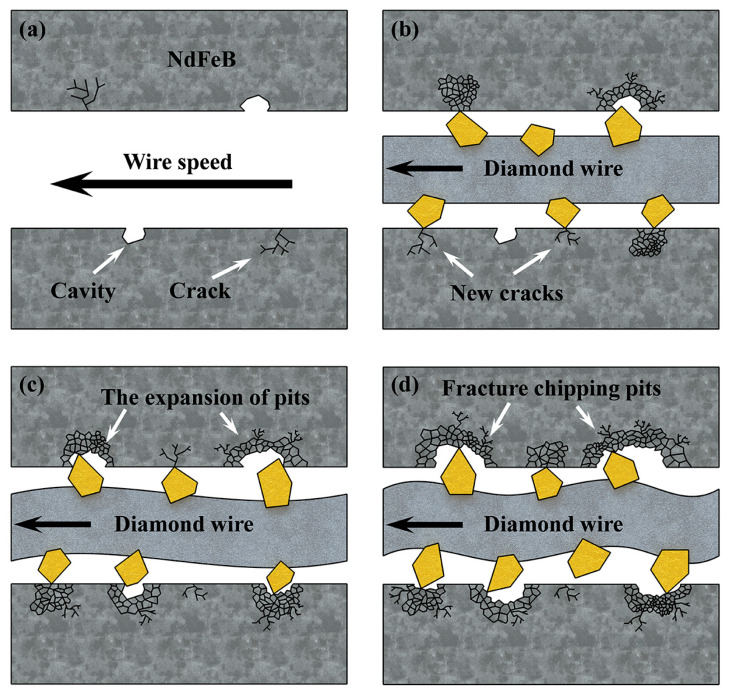
(**a**) Original surface of the NdFeB; (**b**–**d**) are the vicious circle between wire vibration and fracture chipping.

During the sawing process, the impact of abrasives on the surface can loosen the grains and remove them in a brittle mode. In addition, when abrasives strike on the edge of the cavities and fracture chipping pits remain upon brittle removal, the sudden impact induces more cracks that propagate along the grain boundaries since the bonding force of NdFeB is weak. As the cracks constantly spread, more grains gradually become loosened, and when they are impacted again, further fracture chippings can be expected. Moreover, cavities and fracture chipping pits also affect the abrasives’ cutting depth, inducing an abrupt change in force and promoting wire vibration in return. Ultimately, this leads to the formation of more new fracture chipping pits and the expansion of old ones, causing the relationship between wire vibration and fracture chipping to be trapped in a vicious circle, as shown in Figure 15. As for the influence of the feed speed, a higher feed speed provides less time for the interaction between abrasives and the sawed surface at the same location, which can effectively reduce the fracture rate. As a result, the fracture rate on the NdFeB surface is gradually reduced as the feed speed increases from 0.05 mm/min to 0.2 mm/min. However, as the feed speed continues to increase to 0.3 mm/min, the vibration amplitude, as well as the vibration STD, begins to increase, and, as a result, the fracture rate also increases.

It is worth noting that the volume of material removed per unit of time by the diamond wire increases with the feed speed, causing a concomitant increase in the cutting depth of the abrasive. In this regard, Gao et al. [38] proposed Equation (11) for the relationship between the cutting depth of a single diamond abrasive in the ductile regime and the feed speed and the wire speed during the diamond sawing.

(11) $$h_{c}={\left(\frac{10^{\left(-27/8\right)}\cdot {\left(0.3K_{\mathrm{IC}}\right)}^{\left(1/2\right)}\cdot H^{\left(3/8\right)}\cdot \sin\varphi }{0.43\cdot \pi^{\left(3/4\right)}\alpha_{n}\cdot \alpha_{0}{}^{\left(3/8\right)}\cdot \varepsilon^{\left(5/8\right)}{\left(\tan\theta \right)}^{\left(23/12\right)}\cdot {\left(\sin\theta \right)}^{\left(1/2\right)}\cdot E^{\left(7/8\right)}\cdot N}\cdot \frac{v_{f}}{v_{s}}\right)}^{\left(4/9\right)}$$

where *K*_IC_ is the fracture toughness; *H* is the hardness; *φ* is the position angle of the abrasive; *ε* is the tangential load coefficient; *α*_n_ is the indentation coefficient relating to the abrasive shape; *α*_0_ is the dimensionless constant relating to the penetrator shape; *θ* is the half tape angle of the abrasive; *E* is the elastic modulus; and *N* is the dynamic effective abrasive. Equation (11) shows that the cutting depth of the abrasive increases along with the feed speed. Due to the existence of cavities inside NdFeB and the limited intergranular bonding strength between grains, even if the scratching and striking of the NdFeB surface by the wire is reduced as feed speed increases, the impact force caused by the larger cutting depth of the abrasive also induces the NdFeB grain clusters to be peeled off of from the sample surface more quickly, thus forming new fracture chipping pits or enlarging the old ones. This explains the increase in the fracture rate on the surface and the wire vibration amplitude when the feed speed is increased to 0.3 mm/min.

**Figure 16:**
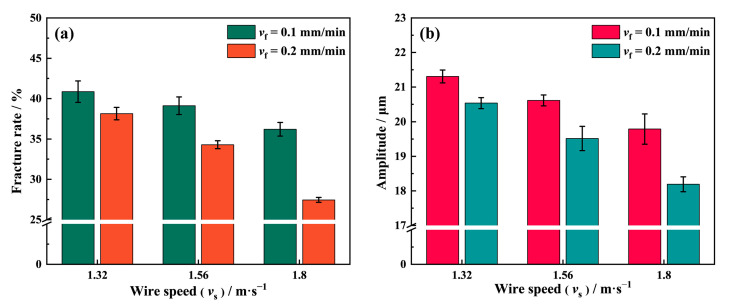
(**a**) Effect of the wire speed on the number of fracture chipping pits; (**b**) the amplitude of the wire vibration at *P* = 0.21 MPa, *v*_f_ = 0.1 mm/min, and *v*_f_ = 0.2 mm/min.

Figure 16a shows the fracture rate on the NdFeB surface sawed at different wire speeds under the condition of *P* = 0.21 MPa. The result shows that the fracture rate at *v*_f_ = 0.2 mm/min is lower than at *v*_f_ = 0.1 mm/min at any given wire speed. The fracture rate gradually decreases when the wire speed increases from 1.32 m/s to 1.8 m/s. It reaches its lowest value, of 27.45%, at *v*_f_ = 0.2 mm/min and *v*_s_ = 1.8 m/s. According to Equation (11), the cutting depth of the abrasive increases as the wire speed decreases, and the formation mechanism of fracture chipping at this time is consistent with that under high feed speed sawing conditions. In addition, when the diamond abrasives scratch brittle materials such as NdFeB, a higher cutting speed and lower cutting depth can also remove the material in the ductile mode. The wire vibration amplitude in Figure 16b shows the surface qualities of the samples with different wire speeds. The amplitude of the wire vibration is the largest, and, correspondingly, the sample surface is the worst in terms of quality, among the three groups at *v*_s_ = 1.32 m/s. This indicates that the vibration of the diamond wire is deeply trapped in the vicious circle shown in Figure 15. In contrast, the cutting depth of the abrasive is smaller at *v*_s_ = 1.8 m/s, which makes it more difficult for the NdFeB grains to be peeled off from the substrate. This results in fewer fracture chipping pits on the sawed surface while keeping the excitation smaller and more stable, meaning that the wire vibration amplitudes are lowest among the three groups at different wire speeds.

**Figure 17:**
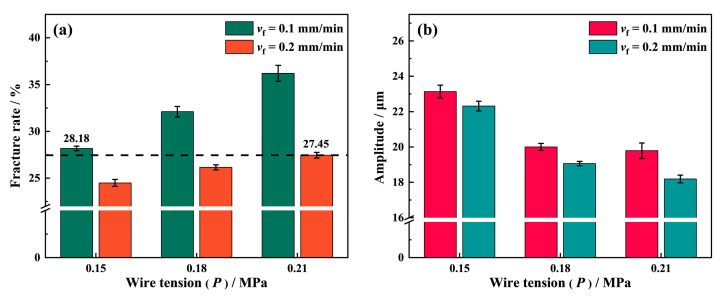
(**a**) Effect of wire tension (*P*) on the number of fracture chipping pits; (**b**) the amplitude of the wire vibration at *v*_s_ = 1.8 m/s, *v*_f_ = 0.1 mm/min and *v*_f_ = 0.2 mm/min.

Wire tension greatly affects the surface quality of NdFeB during sawing with a wire speed of 1.8 m/s. As Figure 17a shows, the fracture chipping pits on the surface of NdFeB samples increases with the wire tension. When the wire tension increases to 0.21 MPa, the fracture rate on the sample surface increases from 28.18% to 36.2% at a feed speed of *v*_f_ = 0.1 mm/min, while it increases from 24.49% to 27.45% at *v*_f_ = 0.2 mm/min. The process of diamond wire sawing is similar to that of grinding. The material removal mechanism in both scenarios involves the the diamond abrasives moving at a high speed to scratch the workpiece, leading to the removal of material in the contact area. However, due to the relatively high length-to-diameter ratio of the diamond wire, it may easily deform when bearing the concentrated force exerted by the diamond abrasives contacting the workpiece, which results in a lower actual cutting depth in terms of the abrasive during sawing than the theoretical cutting depth assuming rigid contact. As the wire tension increases, the deflection and deformation along the lateral direction of the diamond wire gradually decrease under the same concentrated force. Consistent with the study by Wang et al. [28], the vibration amplitude of the diamond wire in Figure 17b decreases in response to the increase in wire tension because the higher tension of the diamond wire lowers the excitation disturbance, which reduces its vibration amplitude in the lateral direction. The diamond wire is much more similar to a rigid body at *P* = 0.21 MPa, causing the actual cutting depth of the diamond abrasive fixed on it to be larger than that at *P* = 0.15 MPa. When the diamond abrasive penetrates into the NdFeB magnet with a large cutting depth, the grains are struck out of the substrate due to the large impact, forming more fracture chipping pits and cracks on the surface. On the contrary, the diamond wire is more flexible at *P* = 0.15 MPa. When the diamond abrasives strike the surface, the diamond wire serves as a buffer to absorb part of the impact energy to reduce the abrasives’ shock to the NdFeB grains. Therefore, it can be clearly seen that the fracture rate at *P* = 0.15 MPa is significantly lower than that with a higher wire tension. Hence, obtaining better surface quality when sawing can be achieved with a lower wire tension. However, due to the feed speed effect, it is worth noting that the fracture rate when sawing at *P* = 0.21 MPa and *v*_f_ = 0.2 mm/min is still lower than when sawing at *P* = 0.15 MPa and *v*_f_ = 0.1 mm/min. Therefore, the sawing efficiency can be doubled under such circumstances, i.e., when the surface quality remains the same.

As is evident from the above discussion, the surface formation process of the diamond wire sawing of NdFeB is complex. The wire tension and wire speed can influence the fracture rate in a simple correlation. However, the influence of the feed speed is much more complicated since the abrasive cutting depth, the kerf width, the dwelling time of the wire, and the wire deflection bow all dynamically change as the feed speed increases. In the future, we will prioritize studying the changes in this complex process when sawing materials with different hardnesses, fracture toughnesses, and crystalline structures are used.

## 4. Conclusions

In this study, the lateral displacement of a diamond wire was monitored by a laser displacement sensor under no-load and sawing conditions. The Savitzky–Golay smoothing algorithm and Fourier filtering algorithm were applied to decompose the lateral motion of the diamond wire. Based on the PV value and the period of saw marks on the NdFeB sawed surface, its formation mechanism was investigated. Moreover, the relationship between the PV value of saw marks and process parameters was also discussed. Finally, the image recognition MATLAB script was used to quantify the sawed surface quality. Then, the effect of different process parameters on the NdFeB surface quality was analyzed by considering the wire vibration and the cutting depth of the abrasive. The main research findings are summarized as follows:

The PV value of saw marks is directly related to the wire speed, the feed speed, and the effective cutting radius (*r*_w_) of the diamond wire. It increases along with the feed speed and decreases conversely with the wire speed. Additionally, there is an optimal feed speed that can match the sawing efficiency to weaken the wire swing, which is also an important variable affecting the PV value, as shown in Equation (10);

Due to the low intergranular bonding strength of NdFeB, the grains are easily peeled off from the substrate by the impact of diamond abrasives during wire sawing. Meanwhile, the process also induces an abrupt change in excitation so that the wire vibrates non-uniformly, which causes more fracture chipping pits. There is also an optimal match between wire vibration and fracture chipping which can obtain a better surface quality. With an increasing wire speed, the cutting depth of the diamond abrasive decreases conversely. This reduces the impact of diamond abrasives on the NdFeB grains, so that they cannot be easily peeled off. It also reduces the excitation exerted on the diamond wire to stabilize the vibration and weaken the vicious circle involving the wire vibration and fracture chipping behavior.

When the diamond wire is under high tension, its deflection deformation weakens so that the cutting depth of the diamond abrasive is larger than that under low tension. Consequently, the fracture rate increases along with the wire tension. However, owing to the feed speed effect, the surface quality sawed at *P* = 0.21 MPa, *v*_f_ = 0.2 mm/min, and *v*_s_ = 1.8 m/s is still better than that sawed at *P* = 0.15 MPa, *v*_f_ = 0.1 mm/min, and *v*_s_ = 1.8 m/s. Therefore, the sawing efficiency can be doubled under such circumstances, i.e., when the surface quality remains the same.

## Figures and Tables

**Table 1:** The composition of the N35 NdFeB magnet.

Element	Content (wt%)	Element	Content (wt%)
Fe	63.54	Gd	0.15
Nd	22.21	Al	0.09
Dy	8.19	Cu	0.07
Co	2.99	Nb	0.06
B	1	Ni	0.04
Pr	0.76	Total	99.1

**Table 2:** Parameters of the diamond wire.

Parameters	Value
Maximum radius *R*_w_ (μm)	115–120
Diamond abrasive particle size *d* (μm)	25–40
Grit distribution density *N* (1/mm^2^)	90
Length of diamond wire *L* (m)	43.2
Distance between guide wheels *l* (mm)	12.5

**Table 3:** Process parameters selection scheme.

Parameters	Value
Driver roller speed (rpm)	220; 260; 300
Wire speed *v*_s_ (m/s)	1.32; 1.56; 1.8
Feed speed of the workbench *v*_f_ (mm/min)	0.05; 0.1; 0.2; 0.3
Wire tension *P* (MPa)	0.15; 0.18; 0.21

**Table 4:** Process parameters of no-load experiments.

No.	1	2	3	4	5	6	7	8	9
*P* (MPa)	0.15			0.18			0.21		
*v*_s_ (m/s)	1.32	1.56	1.8	1.32	1.56	1.8	1.32	1.56	1.8

**Table 5:** Process parameters of sawing experiments.

No.	*v*_s_ (m/s)	*v*_f_ (mm/min)	*P* (MPa)	No.	*v*_s_ (m/s)	*v*_f_ (mm/min)	*P* (MPa)
1	1.8	0.05	0.21	11	1.8	0.2	0.15
2	1.8	0.1	0.21	12	1.8	0.3	0.15
3	1.8	0.2	0.21	13	1.56	0.05	0.21
4	1.8	0.3	0.21	14	1.56	0.1	0.21
5	1.8	0.05	0.18	15	1.56	0.2	0.21
6	1.8	0.1	0.18	16	1.56	0.3	0.21
7	1.8	0.2	0.18	17	1.32	0.05	0.21
8	1.8	0.3	0.18	18	1.32	0.1	0.21
9	1.8	0.05	0.15	19	1.32	0.2	0.21
10	1.8	0.1	0.15	20	1.32	0.3	0.21

**Table 6:** Period of saw marks and the theoretical cutting depth at *P* = 0.21 MPa, *v*_s_ = 1.8 m/s.

Feed Speed *v*_f_	The Period of Saw Marks (*T*_m_)	Theoretical Cutting Depth (*T*_t_)	Relative Error
0.05 mm/min	39.32 μm	40 μm	1.55%
0.1 mm/min	78.91 μm	80 μm	1.38%
0.2 mm/min	157.84 μm	160 μm	1.36%
0.3 mm/min	236.15 μm	240 μm	1.63%

## Data Availability

Not applicable.
